# Medical Legislation

**Published:** 1847-06

**Authors:** 


ARTICLE III.

### MEDICAL LEGISLATION.

There seems to be a disposition on the part of the public, or a part of them at least, to have some legislative action in reference to our profession in Indiana; not to protect themselves from the unprincipled charlatan, for fear such protection might result in a professional monopoly, to the exclusion of those who have *not spent* time, money, and the student's toils to qualify themselves, but to fix a tariff of fees by law to govern us in all cases.

The propriety of this has, year after year, been mooted in our General Assembly, but as yet without any efficient action on the part of that honorable body. Many think it as well to fix the price of corn, flour, and daily labor as the services of physicians.

The following report on the subject, made to the Senate last winter explains itself:

*The Report made by Mr. Bowers, Chairman of the Committee on Agriculture, on the Evil now existing in the shape of Doctors' Bills.*

MR. PRESIDENT:—The committee on agriculture, to whom was referred the petition of Enoch Davis and others, praying the General Assembly to inquire into and remove the evil now extant in the shape of Doctors' bills, have, according to order, had the same under consideration, and have instructed me to report that the committee are sensible of the importance of the subject referred to them, and the magnitude of the evil complained of; that it is a source of no little regret to your committee that they have to acknowledge, not only the existence and magnitude of the evil, but also the daily increase of the same. Various causes may be assigned for the increase of the number of these bills, one of the most prominent of which is the known liberality of the members of the profession, and their unwillingness to force, or even urge, the payment of their bills; depending frequently upon the justice and honesty of their employers to come forward and liquidate their bills, until the physician finds himself embarrassed for the want of those means that a prompt and timely payment of them would have supplied him with. Fotunate would it be if the evil stopped here, but not so: the consciences of the debtors, after a while, become awakened, and, conscious of having neglected their duty, their minds sicken and become diseased, and the body, sympathising with the mind, becomes diseased likewise; the physician then has to be sent for, another bill is added to the former, and thus it goes on, the evil increasing, bill being added to bill, *ad infinitum.*

Your committee are fully of the opinion that the remedy for the evil is far beyond the reach of legislation, and that it remains alone for those upon whom the evil must eventually fall to remedy the same. This can only be done by the prompt payment of the bills, to enable the debtors to effect so desirable an object, the committee on agriculture recommend to the petitioners, and all others similarly situated, early rising in the morning, deep ploughing, a judicious selection of the best seeds, a careful attention to the stock on the farm, a total abstinence from all intoxicating drinks, and to rigidly adhere to the Divine injunction to "render unto Cæsar the things that are Cæsar's;" and with this recommendation, the committee ask to be discharged from the further consideration of the subject.

As is shown by the following, taken from the Boston Medical and Surgical Journal, some of the good people of Connecticut are disposed to seek relief from the same evil:

"*Medical Legislation in Connecticut.*—In the House, May 14th, 'Mr. Howard presented the memorial of John G. Corning and others, praying for a law concerning physicians' fees and prescriptions. The memorial was read. It humbly showed that the healing art had been made too much a mystery for ages, to the injury of morality and giving great facilities for fraud; and the petitioners prayed that a law might be passed directing that all prescriptions shall be legibly written in the English language, that no apothecary shall deliver any medicines without affixing to the bottle or parcel a label in the English tongue, setting forth the contents and mode of mixture; and regulating the fees of physicians. Referred to the committee on patent medicines.'"

We observe that the Ohio Medical Convention, which met at Columbus in May last, has come to the conclusion to solicit the legislature of that State to enact a law in reference to the profession—not to guard against "Doctors' Bills"—but against the impositions of Quackery upon community; and "with a view to giving elevation to the profession in the State, and securing their rights, the following course was adopted:

"*Rosolved*, That a committee of fifteen members of this convention be appointed, whose duty it shall be to frame a proper memorial to be laid before the Legislature of this State at its next session, praying for the enactment of a law regulating the practice of physic and surgery in the State of Ohio.

"*Resolved*, That said committee cause a suitable number of copies of said memorial to be printed and circulated throughout the State, and that every regular physician therein be earnestly requested, and every member of this convention enjoined, to procure the signatures of voters to said memorial, and forward the same to the city of Columbus, on or before the first Monday in December next, directed to the Speaker of the House of Representatives of Ohio.

"*Resolved*, That said committee be further required to draft a bill regulating the practice of physic and surgery, and cause the same to be laid before the legislature at its next session, and that they give their personal attention to the action of said body upon the same until finally disposed of."

